# Clinical applicability and generalizability of an AI-based model predicting surgical site infection after metastatic spine surgery: a correspondence

**DOI:** 10.1097/JS9.0000000000003445

**Published:** 2025-09-08

**Authors:** Qing Chen, Annan Hu, Jinjin Wang, Zeng Lin, Libo Jiang, Jian Dong

**Affiliations:** a Department of Orthopaedic Surgery, Zhongshan Hospital, Fudan University, Shanghai, China; b State Key Laboratory of Molecular Engineering of Polymers, Fudan University, Shanghai, China; c Department of Orthopaedic Surgery, Zhongshan Hospital Wusong Branch Fudan University, Shanghai, China

*Dear Editor,*

We read with great interest the recent article by Cui *et al*, titled “Artificial Intelligence-Based Prediction Model for Surgical Site Infection in Metastatic Spinal Disease: A Multicenter Development and Validation Study” (DOI: 10.1097/JS9.0000000000002806), published in *International Journal of Surgery*^[1]^. The authors present an innovative AI-based model aimed at predicting surgical site infection (SSI) risk in patients undergoing surgery for metastatic spinal disease, addressing a critical gap in clinical practice. This study is particularly commendable for its multicenter design and the application of machine learning to enhance traditional risk models, offering the potential for personalized surgical care. However, we believe there are several aspects of the study that warrant further discussion and constructive critique. In line with the TITAN 2025 guideline on AI transparency^[2]^, a generative AI-based language model was used solely for language polishing and had no bearing on the scientific content.

First, while the reported predictive performance is impressive, we urge caution regarding potential overfitting and the interpretation of performance metrics^[3]^. The model’s Area Under the Curve (AUC) was extraordinarily high (0.986) in the internal cohort, which dropped to 0.848 upon external validation. This substantial delta suggests the possibility that the model may have been overly tailored to the derivation data. The low event rate of SSI (≈6–8%) and the use of numerous predictors (including complex machine learning algorithms) raise the risk of overfitting, where apparent accuracy in development may not fully translate to new patients. We applaud that external validation was performed; however, the external sample was relatively small, and model performance could further regress in broader populations. We encourage the authors to provide more details on any steps taken to mitigate overfitting (e.g., cross-validation, regularization) and to interpret the AUC and other metrics with an understanding of these limitations. As many have noted, most published prognostic models – especially those using AI – are at high risk of bias without rigorous validation. Transparent reporting of model calibration, decision curve analysis, and confidence intervals (which the authors did include) is important to temper optimism with statistical uncertainty. In short, the excellent discrimination is promising but should be confirmed in larger cohorts to ensure it was not a product of modeling the “noise” in the development data.

Second, we are concerned about the inclusion of intraoperative variables in what is ostensibly a preoperative risk tool. The authors identify “surgery time” and “intraoperative blood loss” (implicitly, via the number of spinal segments) among the top predictors of SSI. These factors, while clinically relevant, are only known once surgery is underway or completed. In practical terms, a surgeon cannot know *a priori* how long a case will run or exactly how much blood loss will occur preoperatively. If the goal is early risk stratification – for example, to guide preoperative optimization or prophylactic measures – then incorporating intraoperative parameters limits the tool’s utility at the decision-making stage. This issue has been noted with prior risk scores (e.g., the surgical Apgar score or POSSUM), which require intraoperative data and thus cannot be used for preoperative risk estimation^[4]^. We suggest that the authors clarify the intended use of their model: is it to be applied only after surgery (as a postoperative risk assessment), or do they envision a truly pre-surgery prediction? If the latter, one approach could be to develop a model using only preoperative factors (patient characteristics, tumor factors, planned procedure extent) and perhaps predicted operative variables.

Third, the generalizability of the AI model warrants discussion. The development cohort and external validation cohort were all from tertiary hospitals in the authors’ region. While this multicenter design is a strength, differences in practice patterns, patient demographics, and perioperative protocols across other centers or countries could impact the model’s performance. Indeed, the drop in AUC on external validation suggests some cohort-specific factors at play. Machine learning models, especially those trained on homogeneous data, often struggle to maintain performance when deployed in new settings with distributional shifts^[5]^. We concur with the authors’ call for testing the tool in “diverse patient populations.” Further validation in international or community hospital cohorts would build confidence that the model is broadly applicable. The recent literature emphasizes that even well-performing AI systems are essentially context-bound “rules” that may fail when input data or clinical context changes. We therefore encourage ongoing evaluation: a model update or recalibration might be needed if implemented in a setting with different infection rates or surgical techniques.

Fourth, the authors touch on the clinical implementation of their AI platform, and we wish to expand on the ethical and practical considerations of deploying such a tool. Integrating an AI prediction model into surgical practice is not just a technical step but a socio-technical challenge. Surgeons and care teams must trust and understand the model’s recommendations. We appreciate that the authors identified important predictors (e.g., comorbidity count, tumor type) – this transparency can help clinicians make sense of the risk estimates. We encourage further work on interpretability (e.g., explaining individual predictions) so that the tool can be used in shared decision-making with patients. Moreover, one must consider the ethical implications: prediction models can inadvertently reflect biases in the training data, potentially disadvantaging certain patient subgroups^[6]^.

In summary, Cui *et al.* have presented an innovative application of AI to a difficult surgical problem, and we applaud their contributions. Moving forward, we believe that attention to overfitting risks, a focus on truly preoperative usability, thorough external validation, and careful integration into clinical workflows will greatly enhance the impact of this work. With rigorous prospective evaluation and ethical implementation, such AI-driven tools could meaningfully assist surgeons in identifying high-risk patients and tailoring preventive strategies – ultimately improving outcomes for those with metastatic spinal disease. We look forward to seeing further developments along these lines.

## Data Availability

No new data were generated or analyzed.

## Declarations

AI assistance – a generative AI-based language model was employed solely for language polishing.
